# Genome sequencing, assembly, annotation and analysis of *Staphylococcus xylosus* strain DMB3-Bh1 reveals genes responsible for pathogenicity

**DOI:** 10.1186/s13099-016-0139-8

**Published:** 2016-11-08

**Authors:** Gurwinder Kaur, Amit Arora, Sathyaseelan Sathyabama, Nida Mubin, Sheenam Verma, Shanmugam Mayilraj, Javed N. Agrewala

**Affiliations:** 1 Microbial Type Culture Collection and Gene Bank (MTCC), CSIR-Institute of Microbial Technology, Sector 39-A, Chandigarh, 160036 India; 2 Immunology Laboratory, CSIR-Institute of Microbial Technology, Sector 39-A, Chandigarh, 160036 India

**Keywords:** Coagulase-negative staphylococci, *Staphylococcus aureus*, CDSs, Rapid annotation using subsystem technology (RAST), EzTaxon, Virulence, Disease and defense

## Abstract

**Background:**

*Staphylococcus xylosus* is coagulase-negative staphylococci (CNS), found occasionally on the skin of humans but recurrently on other mammals. Recent reports suggest that this commensal bacterium may cause diseases in humans and other animals. In this study, we present the first report of whole genome sequencing of *S. xylosus* strain DMB3-Bh1, which was isolated from the stool of a mouse.

**Results:**

The draft genome of *S. xylosus* strain DMB3-Bh1 consisted of 2,81,0255 bp with G+C content of 32.7 mol%, 2623 predicted coding sequences (CDSs) and 58 RNAs. The final assembly contained 12 contigs of total size 2,81,0255 bp with N50 contig length of 4,37,962 bp and the largest contig assembled measured 7,61,338 bp. Further, an interspecies comparative genomic analysis through rapid annotation using subsystem technology server was achieved with *Staphylococcus aureus* RF122 that revealed 36 genes having similarity with *S. xylosus* DMB3-Bh1. 35 genes encoded for virulence, disease and defense and 1 gene encoded for phages, prophages and transposable elements.

**Conclusions:**

These results suggest co linearity in genes between *S. xylosus* DMB3-Bh1 and *S. aureus* RF122 that contribute to pathogenicity and might be the result of horizontal gene transfer. The study indicates that *S. xylosus* DMB3-Bh1 may be a potential emerging pathogen for rodents.

**Electronic supplementary material:**

The online version of this article (doi:10.1186/s13099-016-0139-8) contains supplementary material, which is available to authorized users.

## Background

Genus *Staphylococcus* was initially proposed by Ogston [1]. Later on emended by Rosenbach [2]. At present, the genus consists of 49 species and 26 sub-species (http://www.bacterio.net/staphylococcus.html). Till date, 35 species are whole genome sequenced, assembled and annotated some of these are: *Staphylococcus aureus* strain N315 [3], *Staphylococcus carnosus* strain TM 300 [4], *Staphylococcus epidermidis* strain ATCC 1228 [5], *Staphylococcus haemolyticus* strain JCSC 1435 [6], *Staphylococcus lugdunensis* strain HKU09-01 [7], *Staphylococcus pseudointermidus* strain ED 99 [8], *Staphylococcus saprophyticus* strain ATCC 15305 [9], *Staphylococcus warneri* strain SG 1 [10], *Staphylococcus xylosus* strain SMQ-121 [11] and *Staphylococcus cohnii* subsp. *cohnii* [12]. Various members of the genus *Staphylococcus* are commensals and inhabitant of the skin and upper respiratory tracts of mammals [13]. *S. aureus* is the most common species of *Staphylococcus* which causes *Staphylococcal* infections, mainly in the immunocompromised hosts [14]. *S. xylosus* persists as a commensal on the skin of mammals but occasionally in humans. *S. xylosus* is ubiquitous and can be noticed in diverse niches viz. polluted water, fodder, soil surface, etc. [15]. Since, *S. xylosus* is increasingly becoming infectious along with the other staphylococci, therefore it is imperative to explore the genome of this commensal [16–20]. In the public domain totally seven strains of *S. xylosus* have been sequenced at genome level, in which three strains are completely sequenced whereas four strains were having draft genome sequence. Although previous studies in the literature have performed genome annotation and analysis of *S. xylosus*, but a deep analysis on the pathogenicity of this organism obtained from the genomic information through next generation sequencing (NGS) was absent in the literature. Therefore, we generated the draft genome of *S. xylosus* strain DMB3- Bh1 which was isolated from the stool of mouse. Although *S. xylosus* have been reported to be isolated from several sources, we isolated this strain from mouse stool sample during the process of screening several other isolates from mouse stool in order to study mouse gut microbiota. Further a function based comparative genomic analysis of *S. xylosus* strain DMB3-Bh1 with *S. aureus* strain RF-122 and *S. xylosus* strain SMQ-121 were performed using RAST server that revealed genes contributing to pathogenicity in *S. xylosus* strain DMB3-Bh1.

## Results

### Strain identification by 16S rRNA gene sequencing

**Fig. 1:**
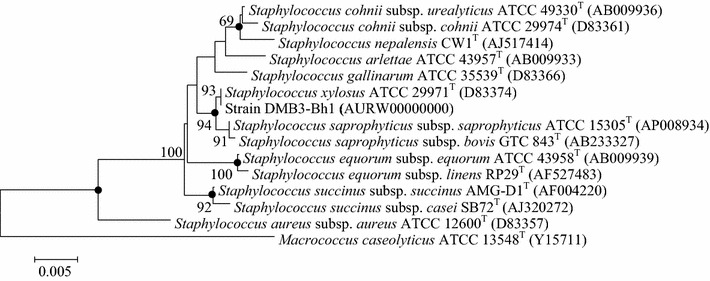
Phylogenetic tree. Neighbour-joining tree based on 16S rRNA gene sequences showing the phylogenetic relationship between *Staphylococcus xylosus* strain DMB3-Bh1 and related members of the genus *Staphylococcus. Macrococcus caseolyticus* ATCC 13548^T^ was used as an out-group. Bootstrap values (expressed as percentages of 100 replications) greater than 50% are given at nodes. Branches recovered in the maximum parsimony and likelihood algorithms are indicated by *filled circles*. GenBank accession numbers are given in the parentheses

An almost complete 16S rRNA gene sequence of the strain (1477 bp) was obtained and phylogenetic analysis showed that the strain DMB3-Bh1 should be assigned in the genus *Staphylococcus*. The strain DMB3-Bh1 showed highest degree of similarity with *S. xylosus* strain ATCC 29971^T^ (100%) followed by *Staphylococcus saprophyticus* subsp. *saprophyticus* strain ATCC 15305^T^ (99.80%), *Staphylococcus saprophyticus* subsp. *bovis* strain GTC 843^T^ (98.66%). The phylogenetic relationship among the species of genus *Staphylococcus* in which strain DMB3-Bh1 formed a separate branch along with *S. xylosus* (Fig. 1). The snp count of the 16S rRNA gene present in the genome of DMB3-Bh1 is 1, its having only a single copy of 16S rRNA gene.

### Genomic features of strain DMB3-Bh1

**Fig. 2:**
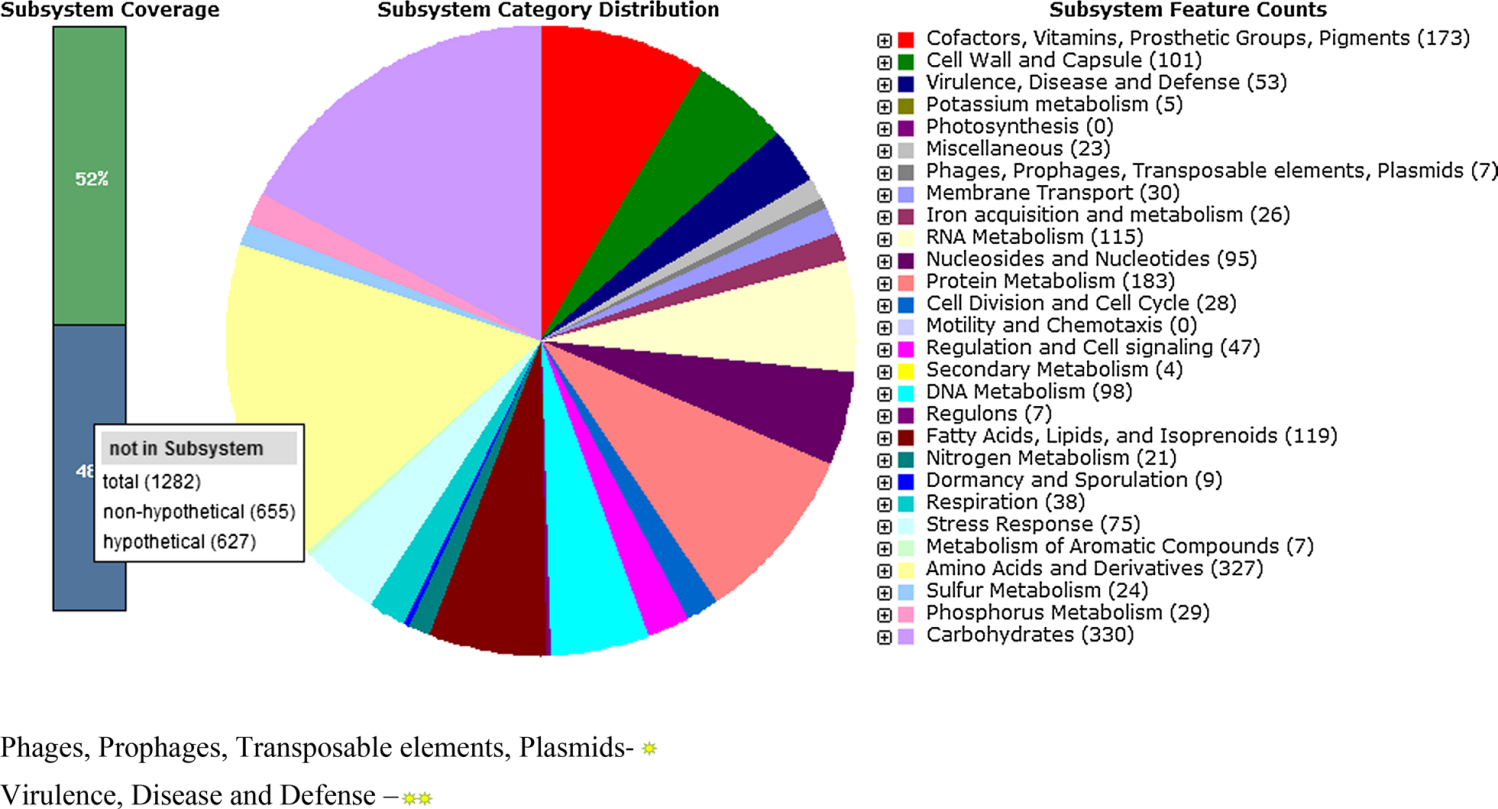
Sub-system distribution. Sub-system distribution of *S. xylosus* DMB3-Bh1 based on RAST annotation

**Fig. 3:**
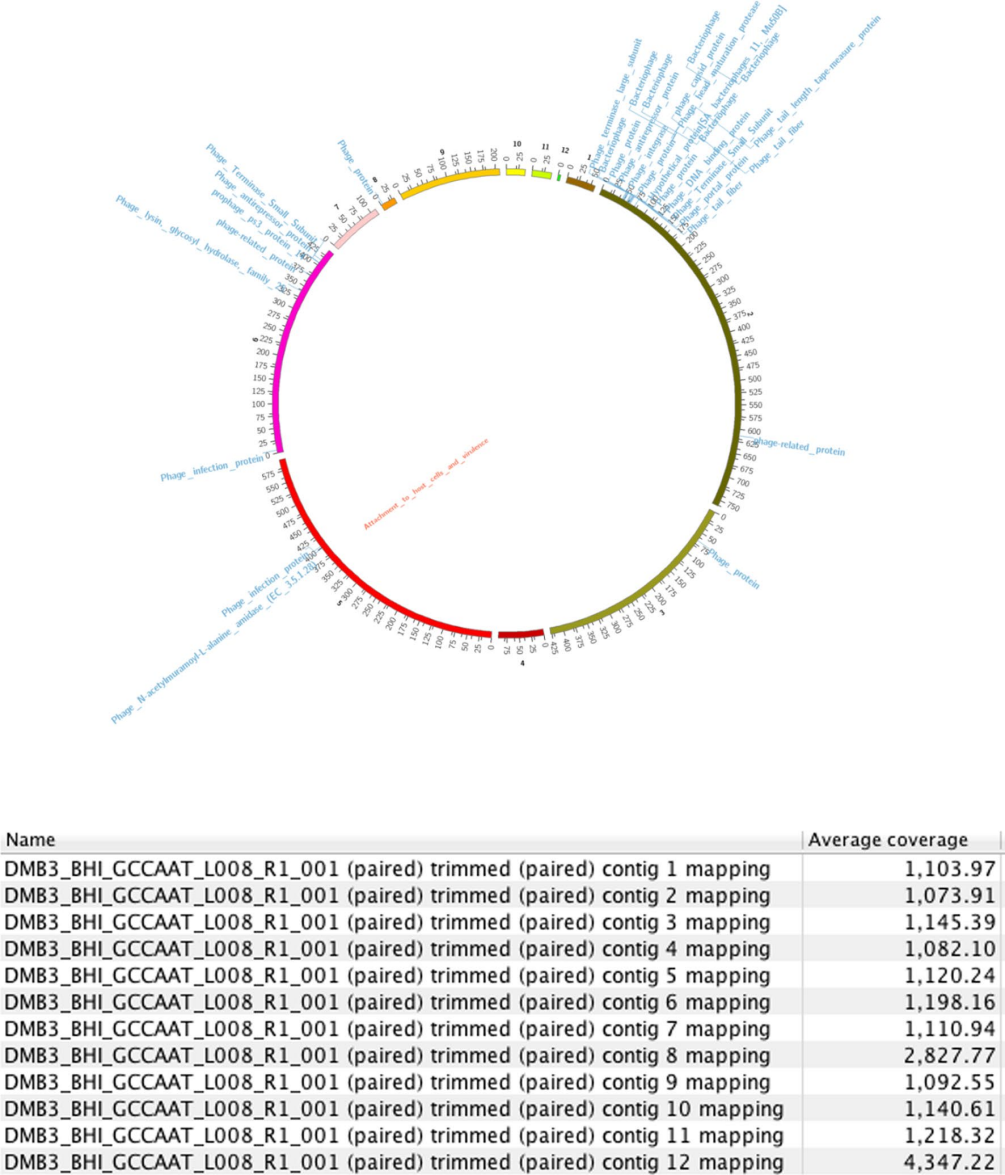
Genome Map. Circular genome map of *Staphylococcus xylosus* DMB3-Bh1 showing the key genes and their regulators. The 12 assembled contigs are presented by different colored ideograms having their base-pair positions depicted at a scale of 1000 units. The coverage of the assembly at each contig is shown in the table. The coverage of the assembly at each base pair can be seen by the *grey colored track*. Annotation descriptors for “virulent” genes (*inner label*: *red*) and “phage” related genes (*outer label*: *blue*) are mapped onto their respective contig positions. As many annotation descriptors occupy neighboring positions on the contigs, the descriptors are stacked to allow better visualization

The draft genome of *S. xylosus* strain DMB3-Bh1 consisted of 28,10,255 bp with G+C content of 32.7 mol%, 2623 predicted CDSs and 58 RNAs. The molar G+C content of the genus *Staphylococcus* ranges from 32.40 to 32.76%. The final assembly contained 12 contigs of total size 28,10,255 bp with N50 contig length of 4,37,962 bp and the largest contig assembled measured 7,61,338 bp. The strain showed forty-nine virulence genes and two genes encoding sub-category adhesions as revealed by RAST annotation server. No OMP’s were detected in the genome of strain DMB3-Bh1. Genome sequencing project information is given in Table 1. Genome Statistics is given in Table 2. Sub-system distribution of *S. xylosus* strain DMB3-Bh1 is depicted in Fig. 2 based on RAST annotation server. The graphical circular map of the genomes is shown in Fig. 3.

**Table 1:** Genome sequencing project information

MIGS ID	Property	Term
MIGS-31	Finishing quality	High quality draft
MIGS-28	Libraries used	Paired end ~330 bp
MIGS-29	Sequencing platforms	Illumina HiSeq 1000
MIGS-31.2	Sequencing coverage	1145.46x
MIGS-30	Assemblers	CLC Bio Workbench v6.0.4
MIGS-32	Gene calling method	Prodigal 1.4, GenePRIMP

	Genbank ID	AURW01000000
	NCBI project ID	PRJNA210599
MIGS-13	Project relevance	Virulence factor, phages

**Table 2:** Genome statistics

Attribute	Value	% of Total
Genome size (bp)	2,810,255	
DNA coding region (bp)	2,364,894	84.15
DNA G+C content (bp)	460,557 + 457,273	32.7
Total genes	2681	84.46
RNA genes	58	0.31
rRNA operons	2	0.16
Protein-coding genes	2623	
Pseudo genes	560	10.47
Genes with function prediction	2121	73.99
Genes in paralog clusters	181	7.28
Genes assigned to COGs	2474	92.28
Genes with transmembrane helices	8	0.28

### Genes involved in virulence, disease and defense

**Fig. 4:**
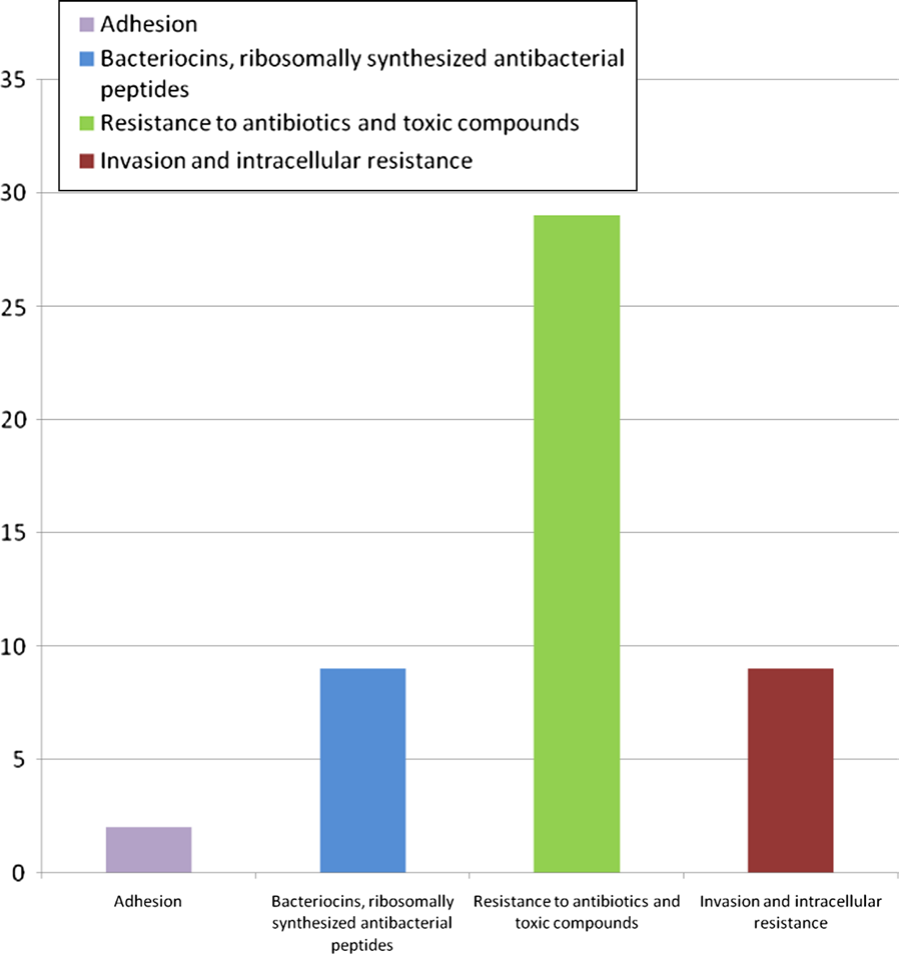
Bar diagram. Genes involved in category virulence disease and defense

Whole genome annotation of *Staphylococcus xylosus* strain DMB3-Bh1 in RAST server revealed a total of 1657 genes. Forty-nine genes encoded for virulence, disease and defense. Some of the genes coding functional proteins are fibronectin binding protein, chaperonin, two component response regulator BceR, bacitracin export ATP binding protein BceA, bacitracin export permease protein BceB, dihydrofolate synthase, folylpolyglutamate synthase, amidophosphoribosyl transferase, acetyl-coenzyme A, carboxyl transferase beta chain, colicin V production protein, tRNA pseudouridine synthase A, copper translocating P type ATPase, MerR family, multidrug resistance protein, membrane component of multidrug resistance system, TetR family regulator protein of MDR cluster, mercuric ion reductase, TcaR arsenical resistance protein ACR3, arsenic efflux pump protein and arsenate reductase (Fig. 4). Numbers of genes associated with the general cluster of orthologous groups (COG) functional categories are given in Table 3.

**Table 3:** Number of genes associated with the general COG functional categories

Code	Value	% age	Description
J	149	6.7	Translation, ribosomal structure and biogenesis
A	0	0.0	RNA processing and modification
K	129	5.9	Transcription
L	115	5.3	Replication, recombination and repair
B	0	0.0	Chromatin structure and dynamics
D	28	1.3	Cell cycle control, mitosis and meiosis
Y	0	0.0	Nuclear structure
V	32	1.5	Defense mechanisms
T	133	6.1	Signal transduction mechanisms
M	119	5.5	Cell wall/membrane biogenesis
N	75	3.5	Cell motility
Z	0	0.0	Cytoskeleton
W	0	0.0	Extracellular structures
U	46	2.1	Intracellular trafficking and secretion, and vesicular transport
O	70	3.2	Posttranslational modification, protein turnover, chaperones
C	142	6.5	Energy production and conversion
G	113	5.2	Carbohydrate transport and metabolism
E	252	11.6	Amino acid transport and metabolism
F	65	3.0	Nucleotide transport and metabolism
H	99	4.6	Coenzyme transport and metabolism
I	44	2.0	Lipid transport and metabolism
P	125	5.8	Inorganic ion transport and metabolism
Q	31	1.4	Secondary metabolites biosynthesis, transport and catabolism
R	243	11.2	General function prediction only
S	161	7.4	Function unknown
–	565	22.5	Not in COGs

### Phages, prophages, transposable elements, plasmids

**Fig. 5:**
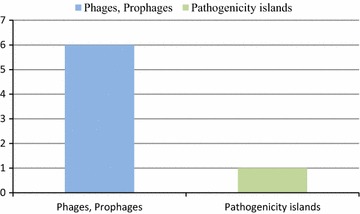
Bar diagram. Genes involved in category phages, prophages, transposable elements, plasmids

A total of 7 protein coding genes were identified in this class. These include, phages DNA binding protein, phage terminase large subunit, phage portal protein, phage tail length tape-measure protein, HNH homing endonuclease and zinc metalloproteinase precursor (Fig. 5).

### Function based comparative genomic analysis

**Fig. 6:**
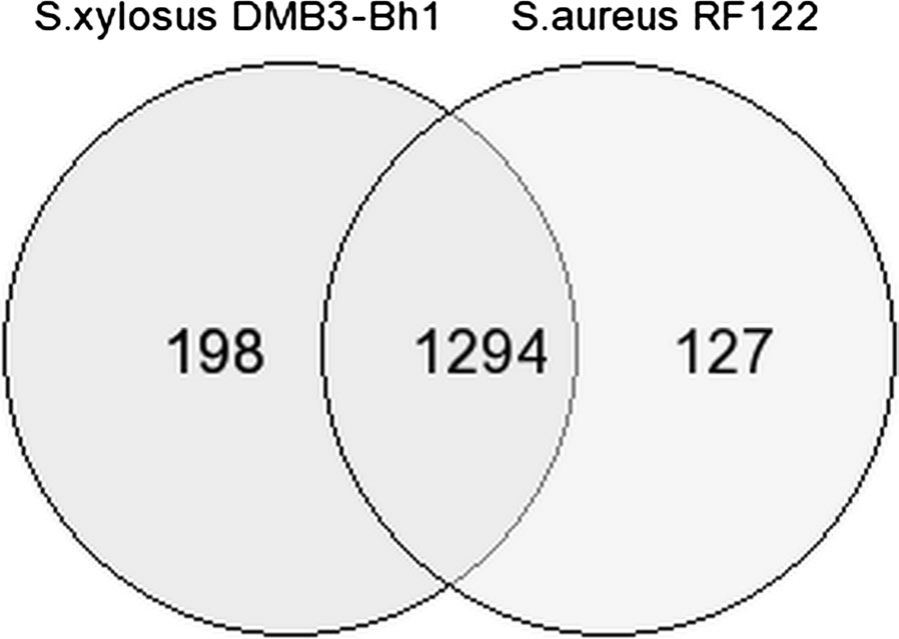
Venn-diagram. Venn-diagram depicting the intersections of protein sets associated with the general COG functional categories

Function based comparative genomic analysis of *S. xylosus* strain DMB3-Bh1, newly sequenced *S. xylosus* strain SMQ-121 and *S. aureus* strain RF122 was achieved with RAST server. A total of 67 protein coding genes were obtained among genomes of *S. xylosus* strain DMB3-Bh1, *S. xylosus* strain SMQ-121 and *S. aureus* strain RF122. Out of the several classes, the prime focus was narrowed down to only two classes (1) virulence, disease and defense; (2) phages, prophages, transposable elements, plasmids in all the three strains. A comparative analysis of these two classes showed 42 genes were present in these three strains. Forty-one genes were common in class virulence, disease and defense, where as one gene was common in class phages, prophages, transposable elements and plasmids. The sequence similarity of the 42 potential virulence factors were determined and it ranges from 30 to 98% for strains RF122/DMB3-Bh1 and 71–100% for strains SMQ121/DMB3-Bh1. Detailed similarity values of the virulence genes among the strains were in Additional file 1: Table S1. Common genes present in both the classes were: Chaperonin (heat shock protein 33), Fibronectin-binding protein, Bacitracin export ATP-binding protein BceA, Bacitracin export permease protein BceB, DNA-directed RNA polymerase beta’ subunit (EC 2.7.7.6), Translation initiation factor 3, Arsenic efflux pump protein, Beta-lactamase (EC 3.5.2.6), Choloylglycine hydrolase (EC 3.5.1.24), cobalt–zinc–cadmium resistance protein, Transcriptional regulator, MerR family. Remarkably five genes contributing pathogenicity were exclusively present in our strain *S. xylosus* strain DMB3-Bh1 and were absent in *S. xylosus* strain SMQ-121. These were Fosfomycin resistance protein FosB, Phage portal protein, Phage tail length tape-measure protein, Phage tail length tape-measure protein and Phage terminase large subunit. The Venn-diagram revealed the number of shared genes in the genome of two closely related strains of genus Staphylococcus i.e. *S. xylosus* strain DMB3-Bh1 and *S. aureus* strain RF122. Out of a total of 1498 COG classes, 1294 were common between *S. xylosus* strain DMB3-Bh1 and *S. aureus* strain RF122. These 1294 COGs corresponded to 19 unique COG classes (B, C, D, E, F, G, H, I, J, L, M, N, O, P, Q, R, S, T, U) (Fig. 6). 13 hypothetical genes found in the genome of strain DMB3-Bh1. One hand, multiple genes can be associated with a single COG class and on the other, a single COG identifier can have multiple class associations. *S. xylosus* strain DMB3-Bh1 had 87 COG identifiers annotated with multiple classes. As these multiple classes are closely related to similar types of biological process, we verified their biological roles by employing the KEGG database [21] and using the corresponding KEGG IDs (KO) to inspect the respective pathways (data not shown).

Further, a comparative genomic analysis strategy was also performed with the virulence genes of *S. aureus* RF122 obtained from VFDB (http://www.mgc.ac.cn/cgi-bin/VFs/genus.cgi?Genus=Staphylococcus), which showed the absence of autolysin, toxins and type-VII secretion systems in genome of *S. xylosus* DMB3-Bh1 compared to *S. aureus* RF122.

## Discussion

This study reports the whole genome sequencing of *S. xylosus* strain DMB3-Bh1 isolated from the mouse stool. The annotated draft genome of strain DMB3-Bh1 was 28, 10,255 bp in size with 2, 623 protein coding sequences. It has 400 subsystems, in which two major classes’ virulence disease and defense; phages, prophages, transposable elements, plasmids were analyzed for genes responsible for infection in mice. This suggests that *S. xylosus* strain DMB3-Bh1 is a potential mouse pathogen and can cause zoonotic diseases. Pathways involved in the pathogenicity and host resistance in *S. xylosus* strain DMB3-Bh1 could be analyzed for novel targets for designing antimicrobial drugs and vaccines. Further, a comparative genomic analysis was performed among *S. xylosus* strain DMB3-Bh1, *S. xylosus* strain SMQ-121 and *S. aureus* strain RF122 that revealed 42 genes were present in all the three strains responsible for infection in the host. This demonstrates that there could be frequent acquisition of virulent factors by *S. xylosus* strain DMB3-Bh1 from *S. xylosus* strain SMQ-121 and *S. aureus* strain RF122, through lateral genetic transfer (LGT) via the mechanisms of transduction, transformation and/or conjugation. The exogenous genetic material can be integrated into the recipient genome through genetic recombination also. In Staphylococcus, phage-mediated conjugation is one of the more common mechanisms of genetic transfer, in which the presence of bacteriophages can increase the adhesiveness of the bacterial cell surface and therefore assist in the conjugative transfer of genetic materials between two organism [22]. There were five genes not present in *S. xylosus* strain SMQ-121 but present in *S. xylosus* strain DMB3-Bh1.This signifies that the strain DMB3-Bh1 has more pathogenic potential as compared to *S. xylosus* strain SMQ-121. The commensal *S. xylosus* is zoonotic agent and can be a threat to human beings [23]. The current study envisages that its genome sequence information will have significant implications in developing novel drug targets drugs and vaccines.

## Conclusions

In the present study we have provided a genomic insight into the genome of *S. xylosus* strain DMB3-Bh1. We were able to map the virulence profile of this organism using in silico approaches. Several putative factors contributing pathogenicity were found in strain DMB3-Bh1 when a comparative pathogenomics approach was employed with other reference strain of genus *Staphylococcus* from publically available databases. Though the data is a preliminary report on the virulence profile of *S. xylosus* strain DMB3-Bh1, such data adds to the repository of virulent pathogens and acts as a building platform for the development of novel therapeutics against emerging pathogens.

## Methods

### Isolation of bacterial strains and growth conditions

**Fig. 7:**
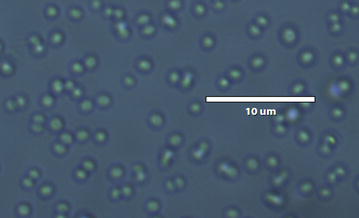
Microscopic image. Microscopic image of strain DMB3-Bh1

*S. xylosus* strain DMB3-Bh1 was isolated from the stool sample of BALB/c. The stool was homogenised in sterile PBS (1X) and centrifuged at 1000 rpm for 2 min to remove debris. Supernatant was serially diluted and plated on tryptone soya agar (TSA, HiMedia, Mumbai, India) and later incubated at 37 °C for 36 h to isolate pure bacterial colonies. Microscopic examination was done to examine cell morphology, motility and sporulation. Cells of strain DMB3-Bh1 are Gram-positive, 0.7–1.0 μm in size (Fig. 7). Different biochemical features were tested. Strain DMB3-Bh1 was positive for urease, nitrate reduction and catalase. Negative for tween 80 and aesculin hydrolysis; oxidase. Acid production was also determined by using different sugars and the strain is positive for glucose, fructose, maltose, xylose, lactose, mannitol, arabinose and negative for cellobiose, galactose, salicin, adonitol, fucose and raffinose. Capable of growing between 20 and 42 °C and tolerant up to 8.0% NaCl. Classification and general features of strain DMB3-Bh1 are accordance with the MIGS recommendations shown in Table 4.

**Table 4:** Classification and general features of *Staphylococcus xylosus* strain DMB3-Bh1 accordance with the MIGS recommendations

MIGS ID	Property	Term	Evidence code
	Current classification	Domain *Bacteria*	[34]
		Phylum *Firmicutes*	[35–37]
		Class *Bacilli*	[38, 39]
		Order *Bacillales*	[40, 41]
		Family *Staphylococcaceae*	[38, 42]
		Genus *Staphylococcus*	[2, 40, 43]
		Species *Staphylococcus xylosus*	[40]
		Strain DMB3-Bh1	Present study
	Gram stain	Positive	IDA
	Cell shape	Coccus	IDA
	Motility	Non-motile	IDA
	Sporulation	Non-sporulating	IDA
	Temperature range	20-45 °C	IDA
	Optimum temperature	37 °C	IDA
	Salinity	3% NaCl	IDA
MIGS-22	Oxygen requirement	Facultatively anaerobic	IDA
	Carbon source	Glucose, fructose	IDA
	Energy source	Fructose, trehalose	IDA
MIGS-6	Habitat	Mouse faecal	IDA
MIGS-15	Biotic relationship	Free living	IDA
MIGS-14	Pathogenicity	Non pathogenic	IDA
	Biosafety level	1	[44]
	Isolation	From mouse faecal sample	IDA
MIGS-4	Geographic location	Chandigarh,India	IDA
MIGS-5	Sample collection time	9th,October 2012	IDA
MIGS-4.1 MIGS-4.2	Latitude–longitude	30.7660° N, 76.7754° E	IDA
MIGS-4.3	Depth	Unknown	IDA
MIGS-4.4	Altitude	About sea level	IDA

Evidence codes—*IDA* inferred from direct assay (first time in publication), *TAS* traceable author statement (i.e., a direct report exists in the literature); If the evidence code is IDA, then the property was observed by one of the authors or an expert mentioned in the acknowledgements

### Strain identification by 16S rRNA gene sequencing

Strain DMB3-Bh1 was identified by 16S rRNA gene sequencing. Genomic DNA was extracted using Zymo Research kit, according to manufacturer’s instructions (Zymo Research Corporation, Irvine, CA). 27f (5′-AGAGTTTGATCCTGGCTCAG-3′) and 1500r (5′-AGAAAGGAGGTGATCCAGCCA-3′) universal eubacterial primers specific for 16S rRNA gene were used for genomic DNA amplification. The amplified product was separated using agarose gel (1%) and finally purified using a QIAquick gel extraction kit (Qiagen, Stockach, Germany). Forward primers, 8-27f, 357f (5′-CTCCTACGGGAGGCAGCAG-3′), 704f (5′-TAGCGGTGAAATGCGTAGA-3′), 1114f (5′-GCAACGAGCGCAACC-3′) and reverse primers 685r (5′-TCTACGCATTTCACCGCTAC-3′), 1110r (5′-GGGTTGCGCTCGTTG-3′) and 1500r (*Escherichia coli* numbering system) were used for amplification of the purified PCR product [24].

### Phylogenetic analysis of strain DMB3-Bh1

Phylogenetic neighbor identification and the computation of pairwise 16S rRNA gene sequence similarities were achieved using the EzTaxon server and alignment was performed using MEGA version 6.0 [25, 26]. The neighbor-joining, maximum parsimony and maximum likelihood algorithms were used to construct phylogenetic trees. Bootstrap analysis was carried out to assess the confidence limits of the branching.

### Genome sequencing, assembly and annotation

The genome of *S. xylosus* strain DMB3-Bh1 was sequenced using a standard run of Illumina HiSeq 1000 sequencing technology at c-CAMP by Next Generation Genomic Facility (Bengaluru, India http://www.ccamp.res.in). The genome of strain DMB3-Bh1 formed a total of 2,91,86,504 paired-end reads [paired distance (insert size) ~330 bp] of 101 bp. The data was preprocessed to trim and remove low quality sequences using CLC Bio Workbench v6.0.4 (CLC Bio, Aarhus, Denmark). A total of 2,90,47,554 high quality, vector filtered reads (~1145 times coverage) were employed for assembly (at word size of 45 and bubble size of 98). The genome coverage was 1145.46× and calculated using formula coverage = (read count × read length)/total genome size. Final draft genome was used for genome annotation employing RAST server [27] and RNAmmer 1.2 server [28]. The graphical circular map of the genomes was made by [29]. COG analysis was performed using the Reversed Position Specific BLAST (RPS BLAST using NCBI COG version 2/2/2011) [30] on the prokaryotic protein database with e-value of 0.001. MS Excel was used to compare the output COG ids between different strains. [31]. The Venn-diagram was made by using [32]. For comparative genomic analysis, the annotated genes were extracted from RAST server into an excel table and manually compared for genomic features. [33].

### Sequence data access

This Whole Genome Shotgun project has been deposited at DDBJ/EMBL/GenBank under the accession AURW00000000. The version described in this paper is the first version, AURW01000000.ftp://ncbi.nlm.nih.gov/genomes/ASSEMBLY_REPORTS/All/GCF_000467225.1.assembly.txt.

## Additional file

**Additional file 1: Table S1.** Sequence similarity of 42 potential virulence factors between strains RF122/DMB3-Bh1 and SMQ121/DmB3-Bh1.

## Abbreviations

CNS: coagulase-negative staphylococci
CDSs: coding sequences
RAST: rapid annotation using subsystem technology
COG: cluster of orthologous groups
