# Supplementary material for: Genome sequencing, assembly, annotation and analysis of Staphylococcus xylosus strain DMB3-Bh1 reveals genes responsible for pathogenicity
Source: Gut Pathog. 2016 Nov 8;8:55. doi: 10.1186/s13099-016-0139-8 (PMC5101723; doi:10.1186/s13099-016-0139-8)
Supplement: Supplementary file 1 — Additional file 1: Table S1. Sequence similarity of 42 potential virulence factors between strains RF122/DMB3-Bh1 and SMQ121/DmB3-Bh1. [file 13099_2016_139_MOESM1_ESM.docx]

**Supplementary Table 1 Sequence similarity of 42 potential virulence factors between strains RF122/DMB3-Bh1 and SMQ121/DmB3-Bh1**

| **S. no** | **Category** | **Name of Gene** | **RF122/**  **DMB3-Bh1 Similarity (%)** | **SMQ121/ DMB3-Bh1**  **Similarity (%)** |
| --- | --- | --- | --- | --- |
|  |  |  |  |  |
| 1 | Virulence, Disease and Defense | Chaperonin (heat shock protein 33) | 81 | 100 |
| 2 | Virulence, Disease and Defense | Fibronectin-binding protein | 30 | 98 |
| 3 | Virulence, Disease and Defense | Bacitracin export ATP-binding protein BceA | 61 | 97 |
| 4 | Virulence, Disease and Defense | Bacitracin export permease protein BceB | 45 | 99 |
| 5 | Virulence, Disease and Defense | Two-component response regulator BceR | 77 | 99 |
| 6 | Virulence, Disease and Defense | Acetyl-coenzyme A carboxyl transferase beta chain (EC 6.4.1.2) | 89 | 99 |
| 7 | Virulence, Disease and Defense | Amidophosphoribosyltransferase (EC 2.4.2.14) | 86 | 100 |
| 8 | Virulence, Disease and Defense | Colicin V production protein | 41 | 98 |
| 9 | Virulence, Disease and Defense | Dihydrofolate synthase (EC 6.3.2.12) | 75 | 99 |
| 10 | Virulence, Disease and Defense | Folylpolyglutamate synthase (EC 6.3.2.17) | 75 | 99 |
| 11 | Virulence, Disease and Defense | tRNA pseudouridine synthase A (EC 4.2.1.70) | 78 | 99 |
| 12 | Virulence, Disease and Defense | DNA-directed RNA polymerase beta subunit (EC 2.7.7.6) | 95 | 100 |
| 13 | Virulence, Disease and Defense | DNA-directed RNA polymerase beta' subunit (EC 2.7.7.6) | 93 | 99 |
| 14 | Virulence, Disease and Defense | LSU ribosomal protein L20p | 97 | 100 |
| 15 | Virulence, Disease and Defense | LSU ribosomal protein L35p | 91 | 100 |
| 16 | Virulence, Disease and Defense | Translation initiation factor 3 | 89 | 100 |
| 17 | Virulence, Disease and Defense | SSU ribosomal protein S12p (S23e) | 98 | 100 |
| 18 | Virulence, Disease and Defense | SSU ribosomal protein S7p (S5e) | 95 | 100 |
| 19 | Virulence, Disease and Defense | Translation elongation factor G | 94 | 99 |
| 20 | Virulence, Disease and Defense | Translation elongation factor Tu | 93 | 99 |
| 21 | Virulence, Disease and Defense | Arsenate reductase (EC 1.20.4.1) | 76 | 71 |
| 22 | Virulence, Disease and Defense | Arsenic efflux pump protein | 73 | 80 |
| 23 | Virulence, Disease and Defense | Beta-lactamase (EC 3.5.2.6) | 52 | 96 |
| 24 | Virulence, Disease and Defense | Choloylglycine hydrolase (EC 3.5.1.24) | 54 | 98 |
| 25 | Virulence, Disease and Defense | Cobalt-zinc-cadmium resistance protein | 22 | 100 |
| 26 | Virulence, Disease and Defense | Transcriptional regulator, MerR family | 34 | 95 |
| 27 | Virulence, Disease and Defense | Zn(II) and Co(II) transmembrane diffusion facilitator | 72 | 99 |
| 28 | Virulence, Disease and Defense | Copper-translocating P-type ATPase (EC 3.6.3.4) | 73 | 98 |
| 29 | Virulence, Disease and Defense | Mercuric ion reductase (EC 1.16.1.1) | 63 | 98 |
| 30 | Virulence, Disease and Defense | PF00070 family, FAD-dependent NAD(P)-disulphide oxidoreductase | 63 | 98 |
| 31 | Virulence, Disease and Defense | Mercuric ion reductase (EC 1.16.1.1) | 63 | 98 |
| 32 | Virulence, Disease and Defense | Membrane component of multidrug resistance system | 62 | 98 |
| 33 | Virulence, Disease and Defense | Multidrug resistance protein [function not yet clear] | 69 | 100 |
| 34 | Virulence, Disease and Defense | TetR family regulatory protein of MDR cluster | 44 | 99 |
| 35 | Virulence, Disease and Defense | DNA gyrase subunit A (EC 5.99.1.3) | 83 | 99 |
| 36 | Virulence, Disease and Defense | DNA gyrase subunit B (EC 5.99.1.3) | 88 | 99 |
| 37 | Virulence, Disease and Defense | Topoisomerase IV subunit A (EC 5.99.1.-) | 81 | 99 |
| 38 | Virulence, Disease and Defense | Topoisomerase IV subunit B (EC 5.99.1.-) | 89 | 99 |
| 39 | Virulence, Disease and Defense | Teicoplanin resistance associated membrane protein TcaA | 50 | 99 |
| 40 | Virulence, Disease and Defense | Teicoplanin resistance associated membrane protein TcaB | 65 | 99 |
| 41 | Virulence, Disease and Defense | Teicoplanin-resistance associated HTH-type transcriptional regulator TcaR | 52 | 100 |
| 42 | Phages, Prophages, Transposable elements, Plasmids | Zinc metalloproteinase precursor (EC 3.4.24.29) | 48 | 99 |
